# Incidence of newly diagnosed diabetes after Covid-19

**DOI:** 10.1007/s00125-022-05670-0

**Published:** 2022-03-16

**Authors:** Wolfgang Rathmann, Oliver Kuss, Karel Kostev

**Affiliations:** 1 Institute for Biometrics and Epidemiology, German Diabetes Center, Leibniz Center for Diabetes Research at Heinrich Heine University, Düsseldorf, Germany; 2 German Center for Diabetes Research, Partner Düsseldorf, München-Neuherberg, Germany; 3 Centre for Health and Society, Medical Faculty and University Hospital Düsseldorf, Heinrich Heine University Düsseldorf, Düsseldorf, Germany; 4 Epidemiology, IQVIA, Frankfurt, Germany

**Keywords:** Coronavirus, COVID-19, Diabetes, SARS-CoV-2

## Abstract

**Aims/hypothesis:**

The aim of this work was to investigate diabetes incidence after infection with coronavirus disease-2019 (Covid-19). Individuals with acute upper respiratory tract infections (AURI), which are frequently caused by viruses, were selected as a non-exposed control group.

**Methods:**

We performed a retrospective cohort analysis of the Disease Analyzer, which comprises a representative panel of 1171 physicians’ practices throughout Germany (March 2020 to January 2021: 8.8 million patients). Newly diagnosed diabetes was defined based on ICD-10 codes (type 2 diabetes: E11; other forms of diabetes: E12–E14) during follow-up until July 2021 (median for Covid-19, 119 days; median for AURI 161 days). Propensity score matching (1:1) for sex, age, health insurance, index month for Covid-19/AURI and comorbidity (obesity, hypertension, hyperlipidaemia, myocardial infarction, stroke) was performed. Individuals using corticosteroids within 30 days after the index dates were excluded. Poisson regression models were fitted to obtain incidence rate ratios (IRRs) for diabetes.

**Results:**

There were 35,865 individuals with documented Covid-19 in the study period. After propensity score matching, demographic and clinical characteristics were similar in 35,865 AURI controls (mean age 43 years; 46% female). Individuals with Covid-19 showed an increased type 2 diabetes incidence compared with AURI (15.8 vs 12.3 per 1000 person-years). Using marginal models to account for correlation of observations within matched pairs, an IRR for type 2 diabetes of 1.28 (95% CI 1.05, 1.57) was estimated. The IRR was not increased for other forms of diabetes.

**Conclusions/interpretation:**

Covid-19 confers an increased risk for type 2 diabetes. If confirmed, these results support the active monitoring of glucose dysregulation after recovery from mild forms of severe acute respiratory syndrome coronavirus 2 (SARS-CoV-2) infection.

**Graphical abstract:**

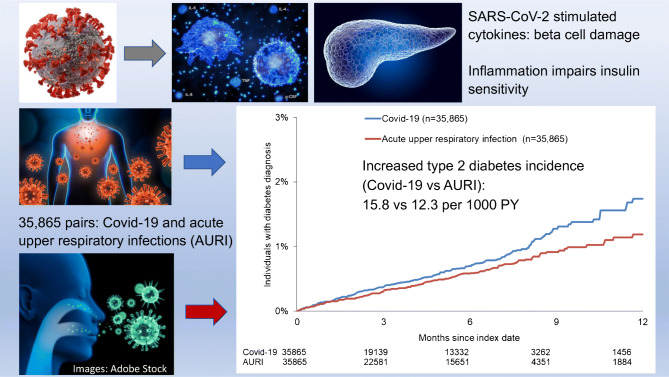

**Supplementary Information:**

The online version contains peer-reviewed but unedited supplementary material available at 10.1007/s00125-022-05670-0.

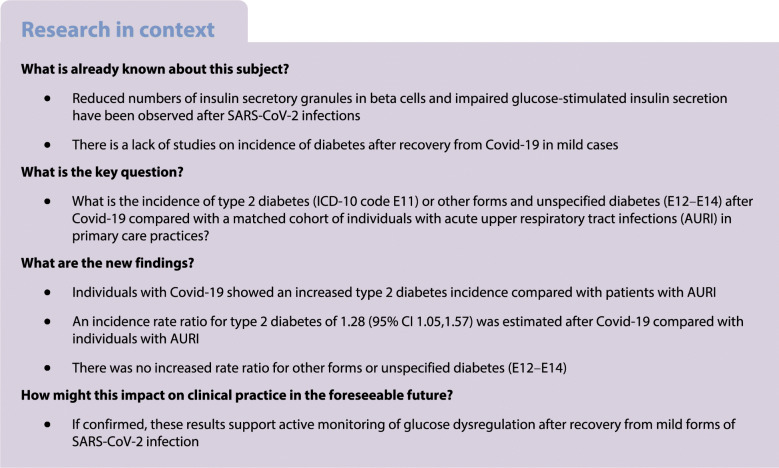



## Introduction

The human pancreas is a target of severe acute respiratory syndrome coronavirus 2 (SARS-CoV-2) [1]. Following SARS-CoV-2 infections, reduced numbers of insulin secretory granules in beta cells and impaired glucose-stimulated insulin secretion have been observed [1]. SARS-CoV-2 may damage beta cells by triggering proinflammatory cytokines [2]. Proinflammatory pathways leading to chronic low-grade inflammation in adipose tissue play an important role in the pathogenesis of insulin resistance and type 2 diabetes [2]. Consequently, new-onset hyperglycaemia and insulin resistance have been reported in patients with coronavirus disease-2019 (Covid-19) without history of diabetes [3]. However, it is unclear whether such metabolic alterations are transient or whether individuals with Covid-19 have an increased future risk of persisting diabetes. The aim of this study was to investigate diabetes incidence after Covid-19 in individuals with mostly mild disease treated in primary care. Individuals with acute upper respiratory tract infections (AURI), which are also frequently caused by viruses (e.g. rhinoviruses), were selected as a non-exposed control group.

## Methods

### Database

The Disease Analyzer (DA) is a computerised healthcare database that comprises a representative panel of physicians’ practices in Germany [4]. The DA assembles drug prescriptions, diagnoses, and basic medical and demographic data directly obtained from the practices’ computer systems. The database has been shown to give valid estimates of the incidence and prevalence of type 2 diabetes, as well as for other chronic diseases [4]. For the present study, we used completely anonymised data from 1171 general practitioners and internists relating to adult patients with one or more visit between March 2020 and January 2021 (*n* = 8.8 million patients) to detect individuals with Covid-19. IQVIA (Frankfurt) is the owner of the database and has given permission to the publication.

### Study populations

The two cohorts studied comprised individuals with newly diagnosed Covid-19 (ICD-10 code U07.1, http://apps.who.int/classifications/icd10/browse/2016/en) or AURI (J00–J06) with index dates of first diagnosis between 1 March 2020 and 31 January 2021. Maximum follow-up was until 31 July 2021. Individuals with a history of Covid-19 (ICD-10 codes U07.1, U08.9, U09.9, U99.0) or a previous diabetes diagnosis (E10–E14) within 365 days prior to the index dates were excluded. In addition, individuals with prescriptions of corticosteroids (dexamethasone, hydrocortisone, methylprednisolone or prednisone) within 30 days after the index dates were excluded. Propensity score matching (1:1) based on sex, age, health insurance coverage, index month and comorbidity (obesity, hypertension, hyperlipidaemia, myocardial infarction, stroke) was performed. Newly diagnosed type 2 diabetes and other forms or unspecified diabetes after the index dates were identified based on ICD-10 codes E11 and E12–E14.

### Statistical analyses

Incidence rates of newly diagnosed diabetes (type 2 and other forms or unspecified diabetes) were calculated according to the person-years method. Kaplan–Meier curves and corresponding logrank tests were estimated for the two diabetes types. Poisson regression models were fitted to obtain incidence rate ratios (IRR) taking account of differential exposure times via offsets. Marginal models were estimated via the generalised estimating equations method to account for correlation of observations within matched pairs. All analyses were performed using SAS 9.4 (SAS Institute, Cary, NC).

## Results

**Fig. 1:**
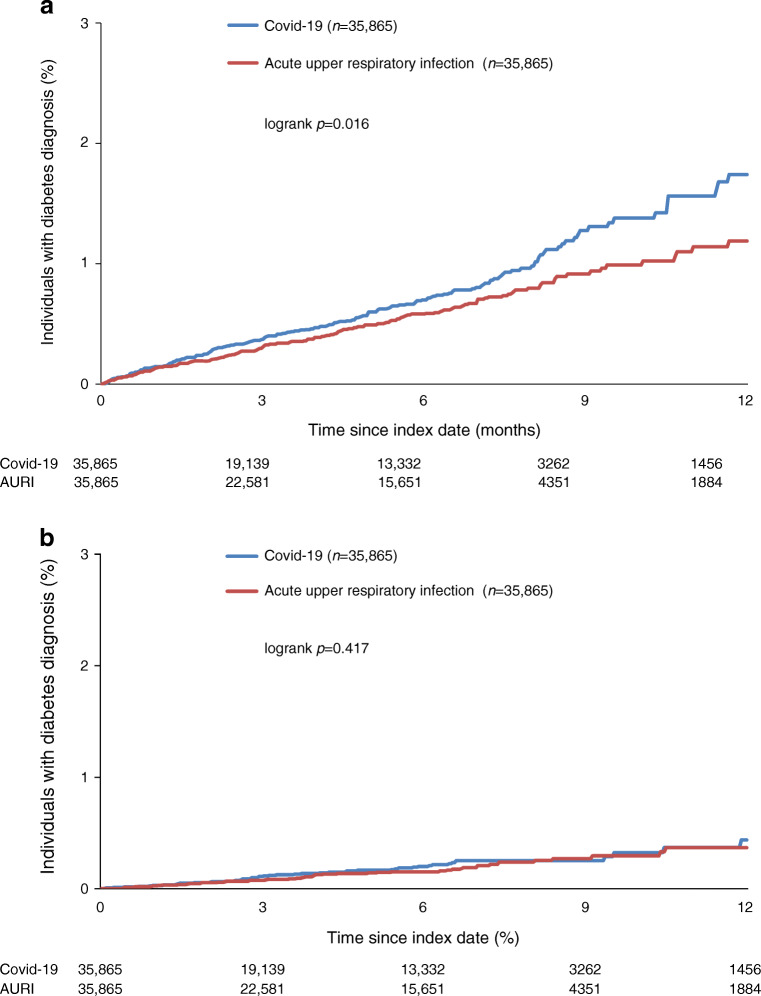
Kaplan–Meier curves for newly diagnosed diabetes in matched individuals with Covid-19 and AURI in the DA primary care database: (**a**) type 2 diabetes (E11), (**b**) other forms of diabetes (E12–14). Propensity score matching (1:1) for sex, age, health insurance coverage, index month and comorbidity (obesity, hypertension, hyperlipidemia, myocardial infarction, stroke)

After applying inclusion and exclusion criteria, there were 35,865 individuals with Covid-19 in the database from March 2020 to January 2021 (electronic supplementary material [ESM] Fig. 1). After propensity score matching, demographic and clinical characteristics were similar in 35,865 AURI controls (ESM Table 1). The mean age was 42.6 years and 45.6% were women.

The follow-up durations (median, IQR, minimum, maximum) were as follows: Covid-19 (119, 0–210, 0, 501 days) and AURI (161, 4–225, 0, 514 days). The numbers of visits during 365 days after index dates were similar in both groups (Covid-19: 6.7 ± 11.7; AURI: 6.8 ± 10.6; means ± SD). During follow-up, the numbers of documented hospitalisations were also comparable in both cohorts (Covid-19: 3.2%; AURI: 3.1%; median number of stays = 1 in both cohorts). Non-steroidal antirheumatics were frequently prescribed in both cohorts at index dates. In individuals with Covid-19, povidone-iodine was more frequently prescribed due to its virucidal activity against SARS-CoV-2, whereas antibiotics were more often used in patients with AURI (data not shown).

Diabetes incidence for type 2 diabetes as well as other diabetes forms was numerically higher in individuals with Covid-19, but only statistically different from controls for type 2 diabetes (Table 1). Overall, 55% of individuals with Covid-19 and 54% of patients with AURI did not receive any glucose-lowering medications at diabetes diagnosis. The first-line glucose-lowering therapies were comparable in both cohorts (Covid-19: metformin 29%; dipeptidyl peptidase-4 [DPP-4] inhibitors 6%; insulin 3%; AURI: metformin 27%; DPP-4 inhibitors: 5%, insulin 3%).

**Table 1:** Incidence and incidence rate ratio of newly diagnosed diabetes after Covid-19 or acute upper respiratory tract infections (AURI)

	Diabetes incidence per 1000 person-years (*n*)		IRR for Covid-19 vs AURI (95% CI)
ICD-10 code	Covid-19 cohort	AURI cohort	
E11 (type 2 diabetes)	15.8 (189)	12.3 (175)	1.28 (1.05, 1.57)
E12–E14 (other forms or unspecified diabetes)	4.3 (52)	3.7 (53)	1.17 (0.80, 1.71)

The Covid-19 (*n* = 35,865) and AURI (*n* = 35,865) cohorts were matched (1:1) for sex, age, health insurance coverage, index month and comorbidity (obesity, hypertension, hyperlipidaemia, myocardial infarction, stroke)

Incidence refers to newly diagnosed diabetes 1–365 days after the index date

IRR were estimated using Poisson regression accounting for different observation durations

Kaplan–Meier curves for the newly diagnosed diabetes types are shown in Fig. 1. The Covid-19 group showed an increased type 2 diabetes incidence, which persisted over the whole period. The diabetes-free survival curves showed a significant difference between the two groups (logrank test: *p*=0.016), whereas no differences for Kaplan–Meier curves were observed for other forms or unspecified diabetes. Diabetes IRRs were increased for type 2 diabetes, but not for other diabetes forms or unspecified diabetes (Table 1). A first sensitivity analysis of type 2 diabetes defined by diagnosis combined with glucose-lowering drug prescriptions yielded an IRR of 1.26 (95% CI 0.93, 1.71). For a second sensitivity analysis, controls with a recorded SARS-CoV-2 test without having ICD-10 code U07.1 within a time interval of 7 days before to 7 days after the index date of AURI diagnosis were selected. After matching (1:1) with individuals with Covid-19 (9823 pairs), incidence rates of type 2 diabetes were 20.5 per 1000 person-years in the Covid-19 group and 13.6 per 1000 person-years in the control group, yielding an IRR of 1.51 (95% CI 1.05, 2.18) (ESM Table 2).

## Discussion

This primary care study found an increased incidence of type 2 diabetes in individuals with Covid-19 after recovery. The strengths of the study are its use of a nationwide primary care database that is representative of diagnoses and drug prescriptions [4]. Second, the sample size was larger than in previous studies, which were mostly hospital-based [5]. Third, recall bias was unlikely because of the use of original data collected in primary care.

The characteristics of individuals with Covid-19 in the present study were largely as expected from national statistics [6]. The median age of all 2.4 million people with Covid-19 in Germany from January 2020 to February 2021 was 44 years, with a slightly larger proportion of women (52%). Of these 2.4 million, 10% were hospitalised, a higher proportion than in the DA, most likely reflecting missing data due to emergency hospitalisations not recorded in the practices. Furthermore, because of the exclusion of individuals with a diabetes history, a healthier sample with milder Covid-19 infections was selected, requiring fewer hospitalisations. Finally, 3.1% of people with Covid-19 in Germany died [6]. Thus, although death is not recorded in the DA, this bias is negligible.

A main limitation of the study is that there was no detailed information on hospitalisations available because the database only includes data obtained by primary care physicians in their clinical practice. Diagnostic data from external specialists and hospitals are only recorded in the database if the primary care physician adds this information. Because of the potential for incomplete medical records, information bias may have been introduced. However, it is most likely that misclassification of exposure and outcome was non-differential, meaning that the errors were the same in the two groups being compared. Furthermore, individuals with undetected diabetes were not included. Transient acute hyperglycaemia, which may have been mistaken as manifest diabetes, could not be ascertained. Individuals with a Covid-19 diagnosis outside of primary care practice (e.g. in hospitals or at Covid-19 test centres) were also not necessarily completely included. Negative SARS-CoV-2 tests were recorded in the practices in only 12,981 (36%) of the AURI group during 365 days after the index date. BMI was not controlled for due to missing data. Furthermore, the number of censored patients due to change of practice or death could not be determined. In the German healthcare system, patients are free to choose a primary care practice. Nevertheless, the vast majority of patients are consistently treated by a single primary care practice. Usually, death of patients is not recorded in German practices. However, the likelihood of short-term mortality is low in patients with mild Covid-19. The incidence of type 1 diabetes was not investigated due to the small number of cases. Finally, the generalisability (external validity) of the data, i.e. the extent to which our results may be applied to other circumstances, requires analysis using separate databases.

The present results from primary care are in line with a retrospective cohort study of hospitalised Covid-19 patients in UK, comprising individuals with more severe disease, higher virus load and greater immune activation [7]. Over a mean follow-up of 140 days, the incidence of new-onset diabetes in 47,780 Covid-19 patients (mean age 65 years; 55% men) was 29 (95% CI 26, 32) per 1000 person-years, yielding a rate ratio of 1.5 (95% CI 1.4, 1.6) compared with matched controls from the general population [7]. Furthermore, merging three databases in the USA (administrative claims, outpatient laboratory testing, inpatient hospital admissions), the hazard ratio for newly diagnosed diabetes in the 6 months after the acute phase of Covid-19 was 2.47 (95% CI 1.14, 5.38) in 266,586 patients infected with SARS-CoV-2 (age 18–65 years) compared with matched controls [8].

Insulin resistance and impaired insulin secretion have been described in individuals without diabetes history who recovered from SARS-CoV-2 infections [3]. Cytokines and TNF-α remain upregulated after remission of Covid-19, which may induce beta cell dysfunction and insulin resistance [3]. Thus, there are plausible mechanisms for a causal relationship between Covid-19 and newly diagnosed type 2 diabetes.

It has been suggested that diagnosis and treatment of post-Covid syndrome require integrated rather than disease-specific approaches [7]. If confirmed, the results of the present study indicate that diabetes screening in individuals who have recovered from even mild Covid-19 should be recommended.

However, there are still a number of unanswered questions for future research. First, it is unclear whether pre-existing diabetes becomes apparent during Covid-19 as a consequence of immunological activation or stress hyperglycaemia [9]. Second, it should be investigated if post-Covid diabetes may be reversed after full recovery [9]. Third, the management of new-onset diabetes after Covid-19 should be evaluated. Diabetes ketoacidosis has been observed in some individuals without known diabetes even months after Covid-19 [10]. Thus, serological testing for diabetes-associated autoantibodies and C-peptide may be indicated in individuals without known risk factors for diabetes after Covid-19. Finally, the risk of hyperglycaemia in individuals with Covid-19 is most likely a continuum, depending on risk factors such as injury of beta cells, an exaggerated proinflammatory response and changes in health behaviour during the pandemic. In particular, by using fixed cut-off points for the definition of incident diabetes, our study may have missed changes in hyperglycaemia risk below these cut-off points. Future studies should investigate the effects of SARS-CoV-2 infections on glucose and HbA_1c_ measurements on a continuous scale.

In conclusion, the present primary care study indicates a temporal relationship between mostly mild Covid-19 and newly diagnosed type 2 diabetes. If confirmed, this study supports the potential relevance of active monitoring of glucose dysregulation after recovery from mild forms of SARS-CoV-2 infection.

## Supplementary information

ESM 1 (PDF 135 kb)

## Data Availability

The Disease Analyzer data are not publicly available due to confidentiality issues. Investigators should contact IQVIA (Frankfurt, Germany) to ask about data availability.

## Abbreviations

AURI: Acute upper respiratory tract infections
Covid-19: Coronavirus disease-2019
DA: Disease Analyzer database
DPP-4: Dipeptidyl peptidase-4
IRR: Incidence rate ratio
SARS-CoV-2: Severe acute respiratory syndrome coronavirus 2
