# Supplementary material for: Incidence of newly diagnosed diabetes after Covid-19
Source: Diabetologia. 2022 Mar 16;65(6):949–54. doi: 10.1007/s00125-022-05670-0 (PMC8923743; doi:10.1007/s00125-022-05670-0)

Electronic Supplemental Material for Rathmann W, et al.

“**Incidence of newly diagnosed diabetes after Covid-19**”

ESM Table 1. Baseline characteristics of people with Coxid-19 and acute upper respiratory tract infections (AURI): Disease Analyzer primary care database

**Variables**	**Covid-19 (N, %)**	**AURI (N, %)**
N	35,865	35,865
Age (mean, SD) years	42.6 (19.1)	42.6 (19.1)
Age 18-30 years	10,816 (30.2)	10816 (30.2)
Age 31-40 years	6500 (18.1)	6500 (18.1)
Age 41-50 years	6133 (17.1)	6133 (17.1)
Age 51-60 years	6631 (18.5)	6631 (18.5)
Age 61-70 years	2932 (8.2)	2932 (8.2)
Age >70 years	2853 (7.9)	2853 (7.9)
Female	16,362 (45.6)	16,370 (45.6)
Private health insurance coverage	1358 (3.8)	1359 (3.8)
Obesity (E66)	1545 (4.3)	1545 (4.3)
Hypertension (I10)	5062 (14.1)	5059 (14.1)
Lipid metabolism disorders (E78)	2772 (7.7)	2775 (7.7)
Stroke (G45, I63, I64)	143 (0.4)	144 (0.4)
Myocardial infarction (I21-I23, I25.2)	72 (0.2)	74 (0.2)
Index month (Covid-19, AURI)		
March 2020	380 (1.1)	402 (1.1)
April 2020	1535 (4.3)	1539 (4.3)
May 2020	855 (2.4)	857 (2.4)
June 2020	641 (1.8)	641 (1.8)
July 2020	869 (2.4)	873 (2.4)
August 2020	1382 (3.9)	1385 (3.9)
September 2020	1377 (3.8)	1381 (3.8)
October 2020	4232 (11.8)	4232 (11.8)
November 2020	8529 (23.8)	8521 (23.8)
December 2020	9742 (27.1)	9725 (27.1)
January 2021	6323 (17.6)	6309 (17.6)

ESM Table 2. Incidence and incidence rate ratio of newly diagnosed diabetes after Covid-19 or acute upper respiratory tract infections (AURI)

	Diabetes incidence per 1000 person-years (n)		IRR for Covid-19 vs AURI (95% CI)
ICD-10 code	Covid-19 cohort	AURI cohort	
E11 (type 2 diabetes)	20.5 (65)	13.6 (50)	1.51 (1.05-2.18)
E12–E14 (other forms or unspecified diabetes)	4.7 (15)	3.7 (14)	1.25 (0.60-2.59)

AURI with a recorded SARS-CoV 2 test without having ICD-10 code U07.1 within a time interval of 7 days before to 7 days after the index date of AURI diagnosis were selected (n=9823). The Covid-19 and AURI cohorts were matched (1:1) for sex, age, health insurance coverage, index month and comorbidity (obesity, hypertension, hyperlipidaemia, myocardial infarction, stroke).

Incidence refers to newly diagnosed diabetes 1-365 days after the index date.

IRR were estimated using Poisson regression accounting for different observation durations

ESM Figure 1 Flow chart of study (Disease Analyzer database)

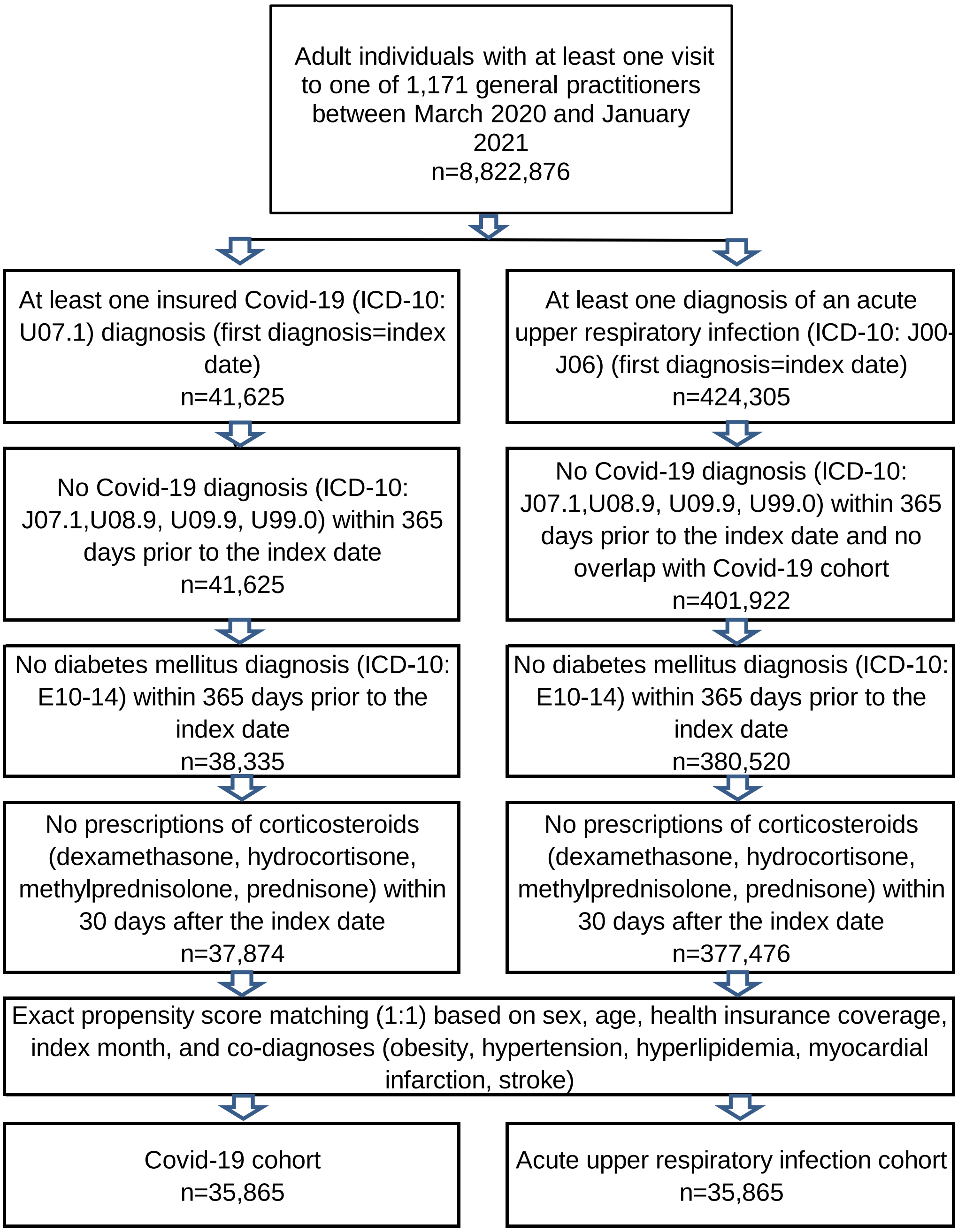

Supplement: Supplementary file 1 — (PDF 135 kb) [file 125_2022_5670_MOESM1_ESM.pdf]
